# Rheumatic Affections of the Stomach

**Published:** 1849-01

**Authors:** R. R. Stone

**Affiliations:** Solon, Ill.


### ARTICLE III.

*Rheumatic Affections of the Stomach.* By R. R. Stone, M. D., of Solon, Ill.

Chronic rheumatic affections of the stomach have been of no uncommon occurrence in this region during the past season, and as they have exhibited themselves by symptoms somewhat mysterious, having the semblance of organic disease, and thereby misleading the practitioner, an investigation of their pathology has become quite interesting. One case of which I will relate as its features are the most prominent:—Sept. 1st, I was called to see Miss S., æt. 23, of that nervous bilious temperament which appears most to predispose to rheumatic affections. I found her with the following symptoms: a peculiar pale, transparent, sallow appearance of the skin, which I considered indicative of schirrus, and I was somewhat confirmed in my convictions, by the statement of her father that his mother died with a cancer of the mamma. Her pulse was small, quick, and wiry; tongue covered with a thick white coating; breath remarkably fœtid; epigastrium extremely tender upon pressure; with frequent paroxysms of severe pain in the part extending into the hypocondriac regions, and to the shoulders; frequently pain in digesting food but not constantly; the reception of food into the stomach always followed by pyrosis, and occasional diarrhœa, alternating with constipation. She appeared to have a species of chill in the after part of the day, followed by a fever and thirst most of the night, and passing off towards morning by a moderate perspiration. In fact, it was a hectic, as anti-periodics seemed to have no power in arresting it.

She was also troubled with amenorrhœa, which I considered merely a consequence of the disease of the stomach. She had been treated by the various Drs. in the vicinity for organic disease, amenorrhœa, dyspepsia, &c., for about three years, but with little or no benefit.

I considered it an organic disease of the stomach, probably of a schirrus nature, or leading to it, and treated the case as such, with cicuta, alteratives, tonics, counter irritations, &c., which partially removed the most urgent symptoms for a time; but they soon failed to give any relief and she grew gradually worse, with sleepless nights from the paroxysms of pain as before named. I was about to give her up as past recovery, when the thought occurred to me (as she appeared to be worse every cold and damp day) that rheumatism might constitute no small portion of the disease. I accordingly prescribed from 2 to 3ʒ vinum colchici per day, when she immediately began to convalesce. With the use of this and the bitter tonics, she rapidly recovered, so that at present she can labor most of the day. The catameniæ have appeared and she now considers herself nearly well. Still, upon exposure to cold and damp weather the pain returns in a mild form, which is almost immediately abated by the exhibition of 40m. vin. colch.

I have found in the treatment of some forms of dyspepsia with those chronic pains in the sides and shoulders, so often complained of in this latitude, that the colchicum is a valuable addition to other therapeutic agents.

November 1st, 1848.

